# I Am More

**DOI:** 10.1017/S1478951525000215

**Published:** 2025-03-24

**Authors:** Zhaohui Su

**Affiliations:** 1 School of Public Health, Key Laboratory of Environmental Medicine Engineering, Ministry of Education, School of Public Health, Southeast University, Nanjing, China; 2 School of Public Health, Southeast University, Nanjing, China

It’s a number
Sure
It’s a mask
Perhaps
It’s a performance
Maybe
It’s a well-earned declaration
Possibly

But really
It’s not
Honestly
There’s more

It’s one reality
For the moment
It’s a presentation
In context
It’s a stage
In transition
It’s a snapshot
In flux

Even truth
Like old age
Has layers upon layers upon layers
Of stories, twists, and turns galore
Alas, there is always something more

I’m turning
I’m transforming
I’m transcending
I’m metamorphosizing
You may think you know me
By my age
By my appearances
By my acquittances
Wait until you see me now

I’m a number
Sure
I’m a mask
Perhaps
I’m a performance
Maybe
I’m a well-earned declaration
Possibly

But really
I’m not
Honestly
I am more

## Data Availability

Data are available upon reasonable request.

